# The Gendered Impacts of COVID-19 amid Agrarian Distress: Opportunities for Comprehensive Policy Response in Agrarian South Asia

**DOI:** 10.1017/S1743923X20000483

**Published:** 2020-07-17

**Authors:** Carly E. Nichols, Falak Jalali, Syed Shoaib Ali, Divya Gupta, Suchita Shrestha, Harry Fischer

**Affiliations:** 1 University of Iowa; 2 University of Iowa; 3 Ambedkar University; 4 Indian School of Business; 5 Southasia Institute of Advanced Studies; 6 Swedish University of Agricultural Sciences

**Keywords:** Care labor, policy, subsistence agriculture, India, Nepal, intra-household bargaining

## Abstract

COVID-19 and its associated disease control measures have greatly altered everyday life. The burden of these challenges has fallen disproportionately on women. Drawing on qualitative inquiry in agrarian north India and Nepal, this research note analyzes how South Asian COVID-19 lockdowns have affected women's labor responsibilities in sometimes surprising ways. We find increased responsibilities for caregiving within the household, substantial stress in responding to food insecurity, and growing expectations to fulfill public roles in disease response measures. However, we also find that the return of male migrants and youth has, in some cases, reduced women's farming responsibilities and created opportunities for household togetherness at a time of great uncertainty. We conclude that more research is needed to examine the nuanced aspects of COVID-19's gendered labor impacts to create comprehensive policy responses to address the multiple and sometimes conflicting effects the lockdown has had on agrarian women's informal labor and well-being.

As COVID-19 and associated movement restrictions upend life around the world, it is well known that the responsibility for responding to these challenges has fallen disproportionately on women (Kamdar 2020; Wenham, Smith, and Morgan 2020). Globally, care work has long been highly feminized (Rai and Waylen 2014), and the shutdowns of schools, businesses, and transport that occurred in response to COVID-19 have intensified and shifted women's roles globally (Wenham, Smith, and Morgan 2020).

In the mid-Himalayan hills of rural India and Nepal, women's unpaid labor has also acted as a shock absorber during the COVID-19 lockdown, which was both strict and sudden. Across South Asia, women's unpaid labor burden is stark, with women averaging six hours per day compared with men's one hour (OECD 2020). These disparities are attributable not only to strictly enforced patriarchal labor roles but also to the fact that large numbers of men and children work and study in cities, so women assume more responsibility for agriculture and livestock. While this is often referred to as the “feminization of agriculture,” South Asian scholars have argued compellingly that a more apt conceptual frame is the “feminization of agrarian distress,” given the poorly remunerative state of agriculture in South Asia along with poor village services and infrastructure (Pattnaik et al. 2018)

In this research note, we draw on qualitative data collected in Nepal and the Indian state of Himachal Pradesh (HP) to explore the complex impacts that COVID-19 and lockdown policies are having on women's labor roles within the context of agrarian distress. Our data suggest while the mass sheltering in place of families significantly *expanded* women's unpaid care work, the presence of returned family members also *diminished* women's agricultural labor burdens and created opportunities for familial togetherness. In highlighting these themes, our aim is to contribute to policy discussions on how looking at relief efforts in South Asia through a feminist lens must entail a more nuanced examination of the ways lockdowns have impacted women within the ongoing crisis of feminized agrarian distress.

## METHODOLOGY

Our data are drawn from qualitative fieldwork conducted from March to June 2020 in 16 villages in Nepal and HP, India. Our sites are socioeconomically diverse, including districts more integrated into urban centers with commercial agriculture (Sirmaur, HP, and Kavre, Nepal), as well as remote areas with higher levels of subsistence agriculture (Chamba, HP, and Ramechhap, Nepal).

Data were collected by trained field assistants from an existing research project that was disrupted by the pandemic. At the lockdown's onset, field staff returned to their homes with detailed instructions to document how the lockdown was affecting their own families, as well as their neighbors and individuals they could contact by phone. Data collection was directed by a series of open-ended questions; assistants were instructed to keep extensive notes throughout the day and reflect on events in daily journals. Participating researchers also conducted 18 remote interviews with research subjects identified by field assistants.

Field assistants were evenly split between men and women, yet all were general category castes (i.e., not low castes) with relatively high education. Since sampling relied on their social networks, privileged groups are more represented in our data. To help limit this bias, assistants were instructed to make explicit efforts to get in touch with marginalized social groups. Despite these limitations, we feel our data provide an invaluable view of the gendered impacts of COVID-19 in mid-Himalayan agricultural communities.

## FINDINGS

Given strong patriarchal gender roles across study sites, it was not surprising that women shouldered additional care work as family members stayed at home. While women had increased responsibilities for cleaning, laundry, and water and fuelwood hauling, the most dramatic impacts related to their role as food preparers.

Women were faced with a double burden of having to feed more people amid a period of unprecedented food scarcity. The lockdown began at the end of March, when monthly rations were almost depleted, and although all impoverished families in HP received two months of advance rations in April, the increased number of mouths to feed caused stress and rationing.^1^ In times of household food scarcity, it is typically the women who eat “last and least” (Lentz, Narayanan, and De 2019), and we heard many reports of similar scenarios across field sites. One Nepali woman stated she used to eat at 9 a.m., but since her household had swelled to 12 people, she was too busy cooking for others, so she now ate alone at noon (Sunita,^2^ Gokulganga, May 25, 2020).

Although the risk of contracting the coronavirus through food supplies is minimal, there were widespread perceptions in the villages that vegetables and rations that came from distant market towns could be holding virus particles. One woman in HP lamented,

The vegetables come from the *mandis* [markets], which are very crowded. Who knows how many hands it has passed through? If one person gets corona the whole village will get it. (Santosh, India, June 9, 2020)

These anxieties around virus contraction, some unfounded, meant that there were marked declines in dietary diversity, and women had to go to extra lengths to secure vegetables within the village. Moreover, women sought to quell household anxiety through more arduous food preparations typically reserved for festivals. This was also something that family members demanded. This was especially true in Nepal, which has higher rates of urban out-migration than India. In one Nepal site, a woman reported feeling highly stressed because her children had returned from Kathmandu and would “get irritated to eat maize every meal,” instead requesting common street food snacks and fruit, which she could only sometimes provide (Sunita, Gokulganga, May 25, 2020).

Women derive much intra-household bargaining power through their roles as food provisioners, and their inability to meet family members demands in a time of scarcity and stress caused them great anxiety. It is critical to note that although most respondents we spoke to had some agricultural lands from which to procure foods, many spoke of marginalized landless households who were suffering because of the shutdown of their daily labor jobs (Birbal, Sirmour, June 14, 2020). Alongside more food preparation, women also had to make more trips to haul water and collect fuelwood for their cookstoves. Because of fears around both the virus and the police, women reported having to haul water from more distant, less utilized water springs or taking circuitous routes to the forest to avoid roadways where police were strictly enforcing lockdown.

### Novel Tasks

Women were also responsible for enacting disease prevention measures and attending to the emotional needs of family members fearful about the pandemic. Most villagers got information about COVID-19 from television news and forwarded WhatsApp messages. Given the dearth of information from state or local officials, our respondents took conservative approaches to disease prevention. These included ensuring their children wore masks and practiced proper hand hygiene as well as preparing heated water throughout the day to ensure good immunity against the virus. As most women operated wood-burning cookstoves, this necessitated more frequent trips to forests for firewood. Finally, with transport shutdown, there was little ability to go to the hospital or clinic so if householders got ill, the women were responsible for caring for them with traditional remedies.

In HP, women also became schoolmasters for children, receiving daily homework assignments via WhatsApp to distribute to their children, whom they also supervised. Women largely reported that their children were unable to focus on or complete school work. This caused them stress as they mourned a lost year of their children's education. Scholars have found that rural parents in India often sustain their own hope for the future through investing in children's education (Jakimow 2016). Perhaps a more enduring yet subtle impact will be on women's diminishing emotional resources to sustain hope through their children's education:

One year of my son's life is lost and as per age he will be behind because in the future if he sits for tests for army, etc., then it will be problematic. . . . Even if they pass the child but he does not know anything that a class 1 student should know and it will cause problem later. Half the year has passed now and doesn't look like the disease will get over anytime soon. (Sunita, Sirmour, May 24, 2020)

Moreover, the ability of children to complete schoolwork was dependent on having a working smartphone in the house, leading to further marginalization of the poor.

### Reduced Agricultural Labor and Togetherness

While women faced significant expansions in the burden of care labor, our data also suggest a reduction in agricultural labor because of the presence of more people at home. For example, in HP, one woman explained there are more people at home so work was completed faster. She said,

Everyone helps in harvesting wheat in the farm. Work that took two days now takes one day. Now we have a lot more free time. Now, we have 3–4 hours of rest [whereas] earlier [we had] had only 1 hour of rest. (Kiran, Sirmour, May 28, 2020)

Experiences similar to Kiran's were observed and reported in every field site. While in some households, girl children were tasked with greater work while boys focused on their studies, most respondents reported that boys would attend to fields and animals while girls assisted with cleaning and cooking.

These findings highlight how agrarian distress has been shouldered by women as men increasingly seek labor work and children pursue studies in both nearby and distant towns. The data resonate with other scholars’ assertion that the so-called feminization of agriculture seen across the global South might be more aptly called the feminization of agrarian distress as women's increased responsibility is not due to empowerment but to other family members’ eagerness to escape the drudgery of smallholder farming. Interestingly, some women also expressed happiness that their families were together, highlighting how the presence of additional household members not only reduced the agriculture work burden but also sometimes improved psychosocial well-being.^3^

## DISCUSSION AND POLICY IMPLICATIONS

While feminist scholarship has long highlighted the exploitation that occurs in the paid and unpaid care economy, there has been slow movement to structure a comprehensive policy approach to address this within South Asia. However, as policies to mitigate COVID-19 have been disproportionately subsidized by women's unpaid care labor, relief efforts offer an opportunity to begin to recognize and compensate women's substantial contributions to the economy. Similarly, the impacts shed light on the ongoing phenomenon of rural distress being shouldered by women.

A comprehensive policy solution would include social support (e.g., cash entitlements) to women as subsidies for unpaid labor but also create a broader investment portfolio into agrarian India to reduce the need for out-migration and reduce the drudgery of women's work. Women's concerns around food, water, and fuel security alongside adequate transport for health service need to be addressed through provisions by state and local government councils. In the medium term, however, we concur with Wenham, Smith, and Morgan (2020) that for any response to be effective, women's voices must be better represented within all relief policies.^4^

This resonates with policy recommendations put forth by feminist collectives both in India (Dasgupta and Mitra 2020) and globally (https://www.feministcovidresponse.com). While COVID-19 and the lockdown have exacerbated gender inequities, they have also highlighted how *during “normal” times* rural women are individually responsible for an untenably broad range of activities in the home and agricultural fields. An effective feminist relief policy, therefore, should not only compensate and acknowledge the central role women are playing in the COVID-response but should seek to learn from these labor dynamics to produce policies that can shift gender inequities across the longer term.

## Footnotes

1. Nepal does not have a national food subsidy program like India, so families there received no rations.

2. Pseudonyms are used for all interviews to protect anonymity.

3. We do not wish to depict an overly sanguine picture, as there is substantive evidence of increased domestic violence across India and other countries throughout the lockdown. However, our data suggest a more complex picture in which many women also sincerely expressed improved well-being as their families were unexpectedly reunited.

4. Wenham, Smith, and Morgan (2020) rightly caution that while women should have a larger say in policy and governance, this need not be another (unpaid) responsibility and proper compensation should be rendered.
